# A study on *Xenorhabdus* and *Photorhabdus* isolates from Northeastern Thailand: Identification, antibacterial activity, and association with entomopathogenic nematode hosts

**DOI:** 10.1371/journal.pone.0255943

**Published:** 2021-08-12

**Authors:** Thatcha Yimthin, Chamaiporn Fukruksa, Paramaporn Muangpat, Abdulhakam Dumidae, Wandee Wattanachaiyingcharoen, Apichat Vitta, Aunchalee Thanwisai

**Affiliations:** 1 Department of Microbiology and Parasitology, Faculty of Medical Science, Naresuan University, Phitsanulok, Thailand; 2 Department of Microbiology and Immunology, Faculty of Tropical Medicine, Mahidol University, Bangkok, Thailand; 3 Department of Biology, Faculty of Science, Naresuan University, Phitsanulok, Thailand; 4 Center of Excellence for Biodiversity, Faculty of Science, Naresuan University, Phitsanulok, Thailand; Universidade Catolica Portuguesa, PORTUGAL

## Abstract

*Xenorhabdus* and *Photorhabdus* are gram negative bacteria that can produce several secondary metabolites, including antimicrobial compounds. They have a symbiotic association with entomopathogenic nematodes (EPNs). The aim of this study was to isolate and identify *Xenorhabdus* and *Photorhabdus* species and their associated nematode symbionts from Northeastern region of Thailand. We also evaluated the antibacterial activity of these symbiotic bacteria. The recovery rate of EPNs was 7.82% (113/1445). A total of 62 *Xenorhabdus* and 51 *Photorhabdus* strains were isolated from the EPNs. Based on *recA* sequencing and phylogeny, *Xenorhabdus* isolates were identified as *X*. *stockiae* (n = 60), *X*. *indica* (n = 1) and *X*. *eapokensis* (n = 1). *Photorhabdus* isolates were identified as *P*. *luminescens* subsp. *akhurstii* (n = 29), *P*. *luminescens* subsp. *hainanensis* (n = 18), *P*. *luminescens* subsp. *laumondii* (n = 2), and *P*. *asymbiotica* subsp. *australis* (n = 2). The EPNs based on 28S rDNA and internal transcribed spacer (ITS) analysis were identified as *Steinernema surkhetense* (n = 35), *S*. *sangi* (n = 1), unidentified *Steinernema* (n = 1), *Heterorhabditis indica* (n = 39), *H*. *baujardi* (n = 1), and *Heterorhabditis* sp. SGmg3 (n = 3). Antibacterial activity showed that *X*. *stockiae* (bMSK7.5_TH) extract inhibited several antibiotic-resistant bacterial strains. To the best of our knowledge, this is the first report on mutualistic association between *P*. *luminescens* subsp. *laumondii* and *Heterorhabditis* sp. SGmg3. This study could act as a platform for future studies focusing on the discovery of novel antimicrobial compounds from these bacterial isolates.

## Introduction

*Xenorhabdus* and *Photorhabdus* are motile, gram-negative rods, facultative anaerobes, non-sporeforming, oxidase-negative, and chemoorganotrophic heterotrophs with respiratory and fermentative metabolism. These bacteria symbiotically inhabit the intestine of the infective juvenile (IJ) stage of entomopathogenic nematodes (EPNs) belonging to the Steinernematidae and Heterorhabditidae families [1]. The IJs of EPNs enter the digestive tract of the insect larvae, penetrate the hemocoel of the insect host, and release the bacteria into the hemolymph. Together, the IJs and bacteria rapidly kill the insect larvae within 24–48 h [2]. Otherwise, the nematodes or bacteria themselves make significant contributions to pathogenesis within the insect [3–5].

*Xenorhabdus* and *Photorhabdus* can produce several secondary metabolites, including insecticidal and antimicrobial compounds, such as benzylideneacetone, phenethylamines, indole, xenocoumacins, 3,5-dihydroxy-4-isopropylstilbene [6, 7], GameXPeptide, xenoamicin, xenocoumacin, mevalagmapeptide phurealipids derivatives, and isopropylstilbene [8]. Several studies on the bioactive compounds of *Xenorhabdus* and *Photorhabdus* against various microorganisms have demonstrated their antibacterial [9], antimicrobial [10], and antiparasitic effects [11].

*Xenorhabdus* and *Photorhabdus* have been isolated from across the world, including Europe, Australia, America, and Asia. Currently, 29 species of *Xenorhabdus* and 20 species of *Photorhabdus* [12] have been reported. Over 90 confirmed species of EPNs [13] have been described from a variety of ecological habitats throughout the world, except Antarctica [14]. In Thailand, six species of *Xenorhabdus*: *X*. *stockiae*, *X*. *miraniensis*, *X*. *ehlersii*, *X*. *vietnamensis*, *X*. *indica*, and *X*. *japonica* [15–18], and three species of *Photorhabdus*: *P*. *luminescens*, *P*. *asymbiotica* subsp. *australis*, and *P*. *temperata* subsp. *temperata have been reported* [8, 17, 19, 20]. Also, at least 11 species of EPNs have been reported from several regions of the country, including *Steinernema siamkayai*, *S*. *surkhetense*, *S*. *websteri* (synonym *S*. *carpocapsae*), *S*. *scarabiae*, *S*. *kushidai*, *S*. *minutum*, *S*. *khoisanae*, *Heterohabditis indica* (synonym *H*. *gerrardi*), *H*. *baujardi* (synonym *H*. *somsookae*), *H*. *bacteiophora*, and *H*. *zealandica* [8, 15, 17, 18, 20–22]. However, there is limited information regarding EPNs and their symbiotic bacteria from Northeastern Thailand.

The objectives of this study to isolate and identify EPNs and their symbiotic bacteria *Xenorhabdus* and *Photorhabdus* from Northeastern Thailand; we also analyzed their phylogenetic diversity. The antibacterial activity of the extracts of the identified *Xenorhabdus* and *Photorhabdus* strains against antibiotic-resistant bacteria was also evaluated using the disk diffusion method, minimum inhibitory concentration (MIC), and minimal bactericidal concentration (MBC). This study will provide information at the molecular level that can assist in taxonomy of *Xenorhabdus* and *Photorhabdus* isolates, and their EPN hosts from Thailand. These bacteria may serve as a resource for discovery a novel bioactive compound.

## Materials and methods

### Collection of soil samples

A total of 1,445 soil samples from 289 soil sites were collected from nine provinces in Northeastern Thailand. All soil sites belonged to public areas and no specific permission was required. For each soil site, five soil samples were randomly taken from an area of approximately 10 m^2^ and at a depth of 10–15 cm using a spade. Approximately 500 g of each soil sample was placed in a plastic bag. Site location, latitude, longitude and altitude, soil temperature, pH, and moisture were recorded. Soil samples were maintained at 25–30°C during transportation to the Department of Microbiology and Parasitology, Faculty of Medical Science, Naresuan University.

### Isolation and identification of entomopathogenic nematodes

The IJs of EPNs were isolated by the baiting technique as previously described [17]. For each soil sample, five larvae of *Galleria mellonella* (greater wax moth) were placed on top of the soil sample stored in a plastic container. Subsequently, the container was covered with a lid, and it was turned upside down to let the larvae move into the soil. It was incubated in dark at 30°C for 5 days. The dead larvae of *G*. *mellonella* were collected from the soil samples and then larval cadavers were placed on a White trap [23] to allow the IJs to emerge. The IJs were collected in a tissue culture flask, cleaned with sterile distilled water, and stored at 15°C.

EPNs were identified by polymerase chain reaction (PCR), which was performed in an Applied Biosystems thermal cycler (Life Technologies, Carlsbad, CA, USA), and sequencing of a partial region of 28S rDNA for *Steinernema* and the internal transcribe spacer (ITS) for *Heterorhabditis*. The primers used were as follows: 539_F (5’GGATTTCCTTAGTAACTGCGAGTG-3’) and 535_R (5’–TAGTCTTTCGCCCCTATACCCTT-3’) for *Steinernema*; 18S_F (5’-TGATTACGTCCCTGCCCTTT-3’) and 26S_R (5’-TTTCACTCGCCGTTACTAAGG-3’) or TW81_F (5’-GTTTCCGTAGGTGAACCTGC-3’) and AB28_R (5’-ATATGCTTAAGTTCAGCGGGT-3’) for *Heterorhabditis*. The PCR reagents and conditions were as described in a previous study [17]. The PCR products were checked on 1.2% agarose gel by electrophoresis.

### Isolation and identification of symbiotic bacteria

The infected dead larvae of the greater wax moth were surfaced sterilized with 95% ethanol before dissection. The hemolymph was collected by a sterile loop, and then streaked onto a nutrient bromothymol blue agar (NBTA). The plate was incubated at 28°C in dark for 4 days. The bacterial isolates (blue or green colonies) were selected and stored in LB broth containing 50% glycerol (v/v) at -80°C.

The genomic DNA of 113 isolates of the symbiotic bacteria was extracted with the Genomic DNA Mini Kit (Blood/Cultured cell) (Geneaid Biotech Ltd., Taiwan) according to the manufacturer’s instructions. A partial sequence of *recA* gene was amplified from the genomic DNA by PCR using forward and reverse primers (5’-GCTATTGATGAAAATAAACA-3’ and 5’-RATTTTRTCWCCRTTRTAGCT-3’) to obtain an 890 bp amplicon (24). The PCR mixture (50μL) consisted of 10 μL of 5X buffer, 7 μL of 25 mM MgCl_2_, 1 μL of 200 mM dNTPs, 2 μL of 5 mM of each primer, 0.5 μL of 5 unit Taq polymerase (Sigma, USA), 2.5 μL of DNA template, and 25 μL distilled water. PCR cycling parameters for the *recA* gene of *Xenorhabdus* were as follows: an initial denaturing step of 94°C for 5 min, followed by 30 cycles of denaturation at 94°C for 1 min, annealing at 50°C for 1 min, and extension at 72°C for 2 min, and a final extension at 72°C for 7 min. PCR parameters for *Photorhabdus* were as follows: an initial denature step of 94°C for 5 min, followed by 30 cycles of denaturation at 94°C for 1 min, annealing temperature of 50°C for 45 s and extension of 72°C for 1.5 min, and a final extension at 72°C for 7 min. The PCR products were checked on 1.2% agarose gel by electrophoresis and purified using the Gel/PCR DNA Fragment Extraction Kit (Geneaid Biotech Ltd., Taiwan).

### PCR for 16S rDNA, *gyrB*, *dnaN gltX*, and *infB*

*recA* analysis revealed that one *Xenorhabdus* (KK9.1_TH) isolate had lower than 96% similarity in the BLASTN search; this isolate was selected for further analysis, and sequencing of its additional nucleotide regions, including 16S rDNA, *gyrB*, *dnaN*, *gltX*, and *infB*, was performed. Primers and PCR reagents used for 16S rDNA, *gyrB*, *dnaN gltX*, and *infB* were as previously described [24, 25]. PCR was performed in a Biometra TOne Thermal cycler (Analytik Jena AG, Jena, Germany). The PCR products were verified on 1.2% agarose gel by electrophoresis.

### Sequence and phylogenetic analysis

The sequencing of the PCR products was done at Macrogen Inc. Service (Korea) (http://www.macrogen.com). The nucleotide sequences were edited and merged with the SeqMan^TM^II software (DNASTAR Inc., Wisconsin, USA). The *recA* sequences of the bacteria from the present study were deposited in the NCBI database under the Genbank accession numbers KY809276 to KY809337, MT160765 to MT160768, and MT158222 for *Xenorhabdus* spp., and KY809338 to KY809388 for *Photorhabdus* spp. The 28S rDNA sequences of *Steinernema* isolates were deposited in the NCBI database under the Genbank accession numbers KY809389 to KY809425, and the ITS sequences of the *Heterorhabditis* isolates were deposited in NCBI database under the Genbank accession numbers KY809426 to KY809468.

The consensus sequences of each species were used for multiple sequence alignment using Clustal W [26] in the MEGA software version 6.0 [27]. Species identification was performed using BLASTN (http://blast.ncbi.nlm.nih.gov/Blast.cgi). Similarity ≥ 97% was considered as the same species. The known nucleotide sequences of EPNs and their symbiotic bacteria in the NCBI database were downloaded and used as the reference species. For EPNs, maximum likelihood (ML) trees of the entire gene (28S rDNA, and ITS) were constructed based on Tamura 3-parameter with 1,000 bootstrap replicates model using MEGA 6.0 software [27]. For symbiotic bacteria, maximum likelihood (ML) trees of the entire gene (16S rDNA, *recA*, *gyrB*, *dnaN gltX*, and *infB*) and the concatenation of truncated sequences of *recA*, *gyrB*, *dnaN gltX*, and *infB* were constructed based on Tamura 3-parameter model using MEGA 6.0 software [27]. Also neighbor-joining trees (NJ) were constructed based on a Kimura 2-parameter with 1,000 bootstrap replicates using MEGA 6.0 software [27]. Bayesian analysis was performed based on Markov chain Monte Carlo method in MrBayes v3.2 [28].

### Preparation of antibiotic-resistant bacteria

Fifteen strains of antibiotic-resistant bacteria, including *Acinetobacter baumannii* (four clinical strains), *Escherichia coli* (two clinical strains), *E*. *coli* ATCC35218, *Klebsiella pneumoniae* (two clinical strains), *K*. *pneumoniae* ATCC700603, *Enterococcus faecalis* ATCC51299, *Pseudomonas aeruginosa*, *Staphylococcus aureus* (two clinical strains), and *S*. *aureus* ATCC20475, were used as the pathogens for testing the antibacterial activity of the extracts of the symbiotic bacteria. These bacteria were streaked on Mueller-Hinton agar (MHA) and incubated at 37°C for 24 h. A single colony was resuspended in 0.85% sodium chloride (NaCl), and the turbidity was adjusted to 0.5 McFarland standards. Then, 100 μL of the bacterial suspension was swabbed on MHA plate for disk diffusion test [29].

### Screening of *Xenorhabdus* and *Photorhabdus* isolates

*Xenorhabdus* and *Photorhabdus* isolates were cultured on NBTA at 28°C in dark for four days. A single colony from each isolate was transferred to a 15-ml tube containing 5 mL of LB broth and incubated at room temperature for 48 h under shaking conditions. A paper disk (6 mm with diameter) with a 20 μL drop of the whole cell culture was placed on MHA plated with antibiotic-resistant bacteria. The plates were placed in an incubator at 37°C for 24 h. The inhibition zone (clear zone) was checked and measured (millimeter). The most effective isolates of *Xenorhabdus* and *Photorhabdus* were selected for crude compound extraction.

### Bacterial extracts

A single colony of *Xenorhabdus* and *Photorhabdus* on NBTA medium was inoculated to 1000 mL flask containing 500 mL of LB. The culture flask was shaken at 180 rpm for 72 h. The bacteria cultured was added with 1000 mL ethyl acetate and mixed well. All solvents were removed from bacterial extracts by a rotary vacuum evaporator (Buchi, Flawil, Switzerland). Dimethyl sulfoxide (DMSO) was added to bacterial extracts to make a final concentration of 500 mg/mL and stored at -20°C until used.

### Disk diffusion method

Cultured drug resistant bacteria were spread on MHA agar. A sterile 6 mm disc was put onto MHA agar plate and then 10 μL of each bacterial extract was dropped onto a sterile disc. Negative control was DMSO and Positive control was antibiotic disks. The plates were incubated at 37°C for 24 h. The inhibition zone was measured in millimeter. The most effective results of bacterial extracts were further evaluated by MIC and MBC.

### MIC and MBC assays

Bacterial extracts were diluted in two-fold serial dilutions in a 96-well micro titer plate. The suspension of drug resistant bacteria (1 × 10^8^ cell/ml) was added into each well and mixed well. Cultured drug resistant bacteria, cultured drug resistant bacteria mixed with DMSO, and sterile Mueller-Hinton (MH) broth were used as controls. Plates were incubated at 37°C for 24 h. No visible growth of drug resistant bacteria in the well was considered as MIC. In addition, 10 μL from each well from the MIC assay was dropped onto MHA plates. The plates were then incubated at 37°C for 24 h. The lowest concentration of bacterial extract without growth of drug resistant bacteria was considered as MBC.

## Results

### Isolation of EPNs

A total of 1,445 soil samples from 289 sites were collected from the Northeastern region of Thailand, including Kalasin [30], Khon Kaen, Chaiyaphum, Nakhon Ratchasima, Maha Sarakham, Loei, Nong Khai, Nhong Bua Lamphu, and Udon Thani provinces. The recovery rate of EPNs was 7.82% (113/1,445) of the total soil samples collected. We isolated 62 strains belonging to *Xenorhabdus* spp. and 51 strains belonging to *Photorhabdus* spp. from the EPNs (Table 1). Most of the soil samples were positive with only one of the two genera of EPNs (*Steinernema* spp. and *Heterorhabditis* spp.). In contrast, few soil samples (two samples from Maha Sarakham province and one sample from Nong Khai province) were positive with both *Steinernema* and *Heterorhabditis*. Most of the EPNs were isolated from loam, and the mean pH, temperature, and moisture of the soil samples were 6.6, 28.4°C, and 1.5%, respectively (Table 2). These soil parameters were not significantly different between soil samples with and without EPNs (Mann-Whitney test).

**Table 1:** Number of symbiotic bacteria isolated from soil samples from the Northeastern region of Thailand.

Province	No. of soil sites	No. of sampling sites with EPNs (%)			No. of soil samples	No. of soil samples with EPNs (%)		
		*Xenorhabdus*	*Photorhabdus*	Total		*Xenorhabdus*	*Photorhabdus*	Total
Kalasin	24	6	3	9 (37.50%)	120	9	3	12 (10.00%)
Khon Kaen	29	5	5	10 (34.48%)	145	5	6	11 (7.58%)
Chaiyaphum	60	9	8	17 (28.33%)	300	11	11	22 (7.33%)
Nakhon Ratchasima	66	11	12	23 (34.85%)	330	17	14	31 (9.39%)
Maha Sarakham	26	3	6	9 (34.62%)	130	4	7	11 (8.46%)
Loei	22	2	0	2 (9.09%)	110	2	0	2 (1.82%)
Nong Khai	20	2	2	4 (20.00%)	100	3	2	5 (5.00%)
Nong Bua Lamphu	22	7	5	12 (54.55%)	110	7	5	12 (10.91%)
Udon Thani	20	3	3	6 (30.00%)	100	4	3	7 (7.00%)
**Total**	289	48 (16.60%)	44 (15.22%)	92 (31.82%)	1,445	62 (4.29%)	51 (3.53%)	113 (7.82%)

**Table 2:** pH, temperature, and moisture content of the soil samples (n = 1,445) in the presence and absence of EPNs.

Soil parameter	Soil with EPNs		Soil without EPNs		*P*-value (Mann-Whitney test)
	(n = 113)		(n = 1,332)		
	Range	Mean	Range	Mean	
pH	5.2–7.0	6.6	3.4–7.5	6.6	0.2144
Temperature (°C)	25–34	28.4	22–39	28.6	0.8401
Moisture (%)	0.0–8.0%	1.5%	0.0–8.0%	1.7%	0.7039

### Identification and phylogenetic tree of *Xenorhabdus* and *Photorhabdus* isolates

Sixty-two isolates belonging to the genus *Xenorhabdus* were identified using BLASTN search of partial sequence of the *recA* gene. Most *Xenorhabdus* isolates (n = 60) were identified as *X*. *stockiae* (97–100% identity). One isolate (bKK26.2_TH) was identified as *X*. *indica* (97% identity), but the remaining one isolate (bKK9.1_TH) was unidentified due to low similarity of its *recA* sequence to that of *X*. *thuongxuanensis* (95% identity) and *X*. *eapokensis* (94% identity). To confirm the species of *Xenorhabdus* bKK9.1_TH, five additional genes (16S rDNA, *gyrB*, *dnaN*, *gltX*, and *infB*) were amplified and sequenced. Nucleotide sequences of four genes revealed high similarity to that of *X*. *eapokensis*: 16S rDNA and *infB* (99% identity), *dnaN* and *gltX* (98% identity). *gyrB* sequence of *Xenorhabdus* bKK9.1_TH showed low similarity with that of *X*. *eapokensis* (90%).

**Fig 1:**
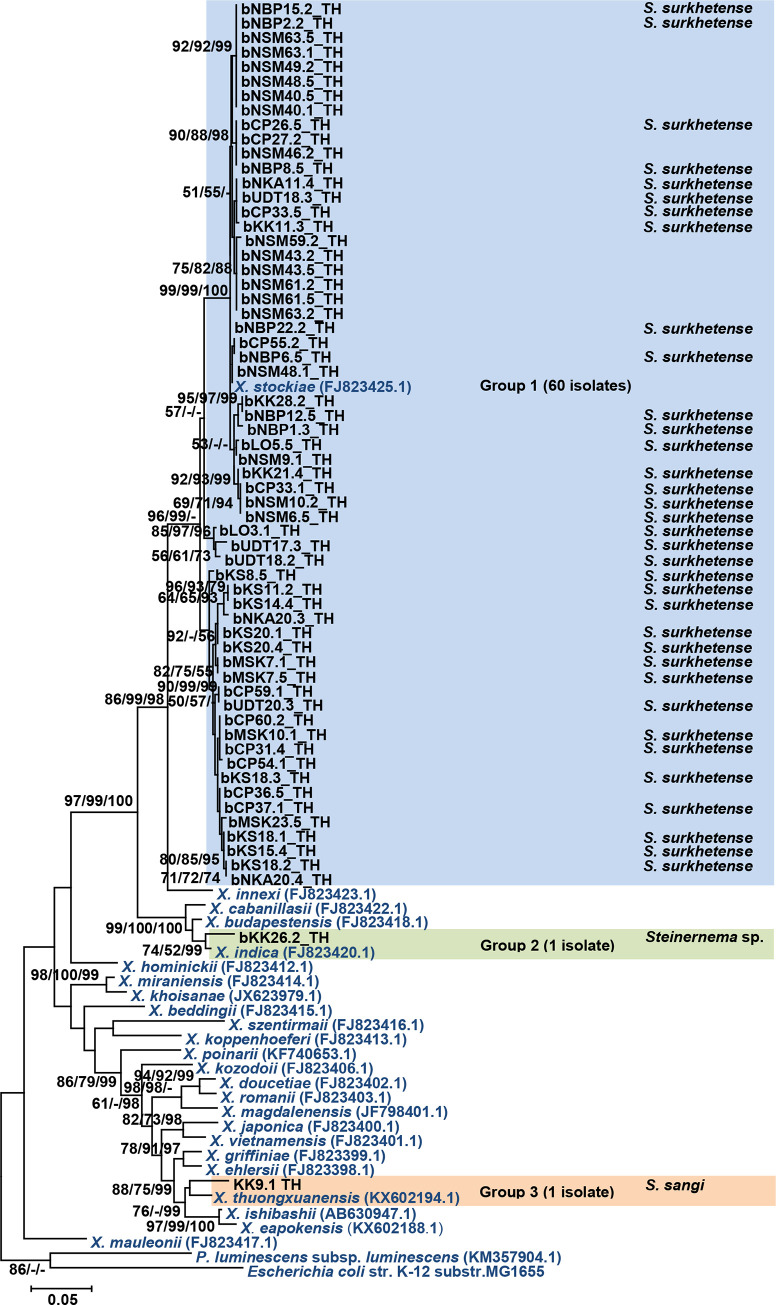
Maximum likelihood tree of 62 *Xenorhabdus* isolates (black bold letter) based on *recA* gene (588 bp) compared with *Xenorhabdus* strains downloaded from GenBank. *Escherichia coli* was used as an out-group. Bootstrap values are reported out of 1000 replicates. The numbers shown above the branches are support values of Maximum likelihood/Neighbor-joining/Bayesian posterior probabilities for clades supported above the 50% level. The bar indicates 5% sequence divergence. The EPN species from which they were isolated are also shown.

**Fig 2:**
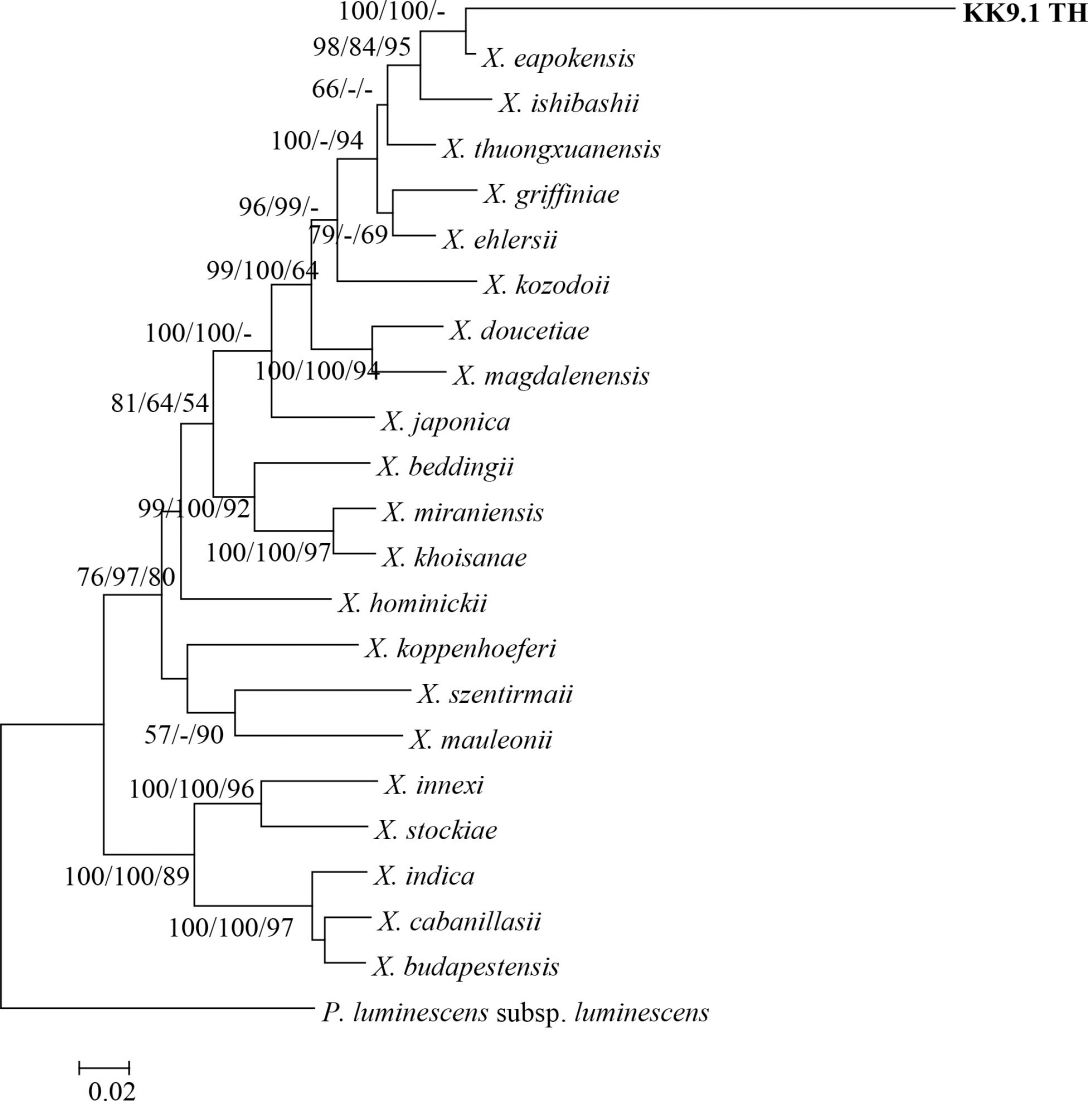
Maximum likelihood tree of concatenated sequences of *Xenorhabdus* sp. (KK9.1_TH) (shown in bold letter) based on truncated *recA* (588 bp), *gyrB* (846 bp), *dnaN* (828 bp), *gltX* (1,057 bp), and *infB* (1,052 bp) compared with the sequences of *Xenorhabdus* strains from GenBank. *P*. *luminescens* subsp. *luminescens* is included as an out-group. Bootstrap values are reported out of 1000 replicates. The numbers shown above the branches are support values of Maximum likelihood/Neighbor-joining/Bayesian posterior probabilities for clades supported above the 50% level. The bar indicates 2% sequence divergence.

The ML tree derived from all the sequences of *recA* among the Thai *Xenorhabdus* isolates and reference strains from GenBank database is shown in Fig 1. The Thai *Xenorhabdus* isolates were distributed in three groups. Group 1 was the majority group (60 isolates), which was closely related to *X*. *stockiae*. Group 2 contained one isolate (bKK26.2_TH), which was closely related to *X*. *indica*. Group 3 also contained one isolate (bKK9.1_TH), which fell in the clade of *X*. *thuongxuanensis*, *X*. *ishibashii*, and *X*. *eapokensis*. The ML tree derived from 16S rRNA, *gyrB*, *dnaN*, *gltX*, and *infB* genes are shown in S1–S5 Figs. The ML tree of concatenation of the five truncated genes (*recA*, *gyrB*, *dnaN*, *gltX*, and *infB*) is shown in Fig 2. All phylogenetic trees supported that *Xenorhabdus* bKK9.1_TH was closely related to *X*. *eapokensis*.

**Fig 3:**
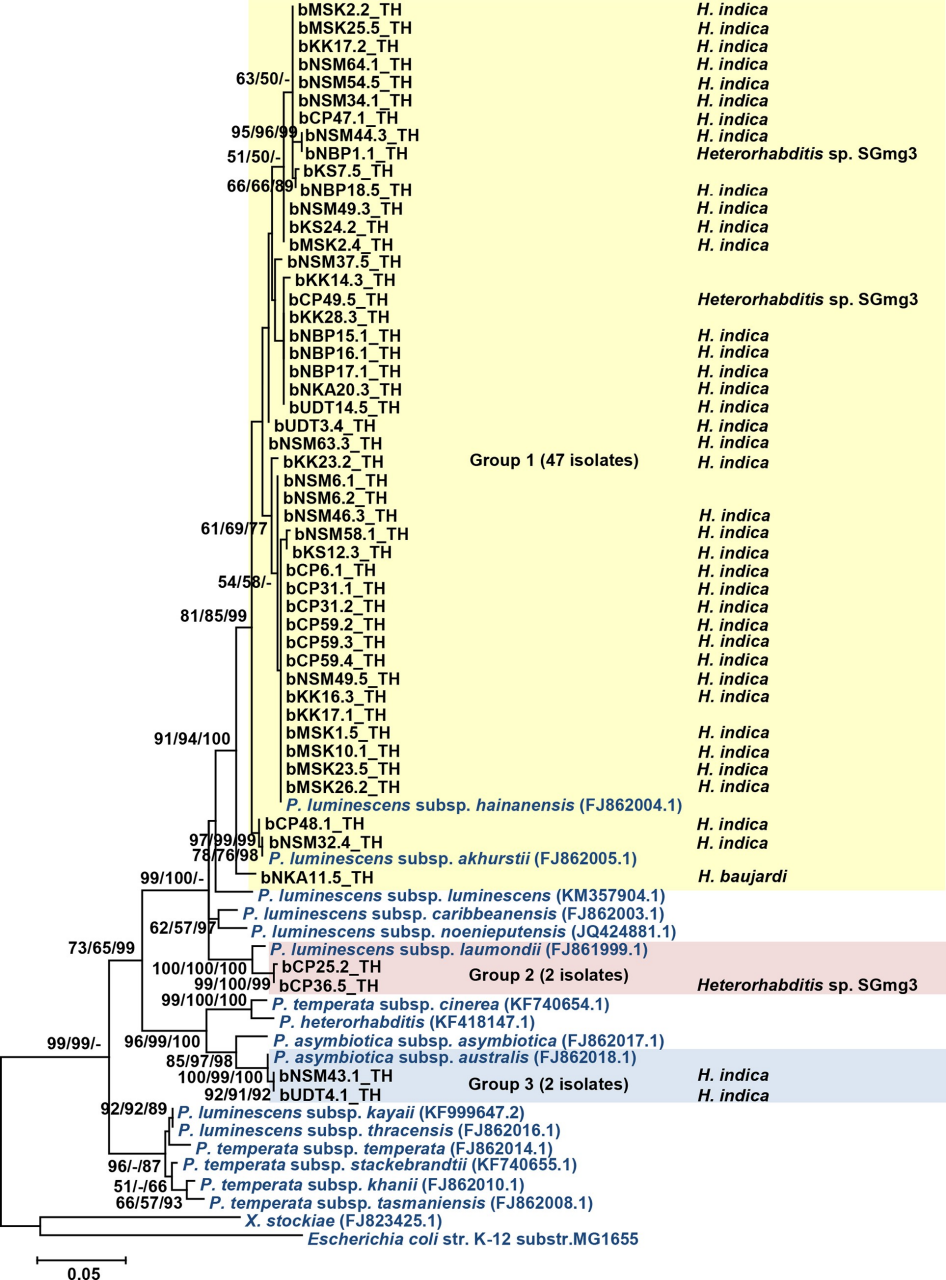
Maximum likelihood tree of 51 *Photorhabdus* isolates (black bold letter) based on the 588 bp region of *recA* gene compared with that of the *Photorhabdus* strains from GenBank. *Escherichia coli* was included as an out-group. Bootstrap values are reported out of 1000 replicates. The numbers shown above the branches are support values of Maximum likelihood/Neighbor-joining/Bayesian posterior probabilities for clades supported above the 50% level. The bar indicates 5% sequence divergence. The EPN species from which they were isolated are also shown.

Fifty-one isolates of *Photorhabdus* were identified using BLASTN search of partial sequences of the *recA* gene. Twenty-nine isolates were identified as *P*. *luminescens* subsp. *akhurstii* (97–100% identity) and 18 isolates were identified as *P*. *luminescens* subsp. *hainanensis* (98–100% identity). Two isolates were identified as *P*. *asymbiotica* subsp. *australis* (99–100% identity). The remaining two *Photorhabdus* isolates were identified as *P*. *luminescens* subsp. *laumondii* (98% identity). ML analysis of 51 *recA* sequences of *Photorhabdus* distributed the isolates in three groups. Group 1 contained 47 isolates closely related to *P*. *luminescens* subsp. *akhurstii* and *P*. *luminescens* subsp. Group 2 contained two *Photorhabdus* isolates, which were closely related to *P*. *luminescens* subsp. *laumondii*. Group 3 contained the remaining two isolates of *Photorhabdus*, which were most closely related to *P*. *asymbiotica* subsp. *australis* (Fig 3).

### Identification and phylogenetic tree of entomopathogenic nematodes

A total of 113 EPNs were isolated from the soil samples. EPNs (80 isolates) were identified using BLASTN search of a partial sequence of 28S rDNA for *Steinernema* and internal transcribed spacer for *Heterorhabditis*. The remaining 33 isolates of EPNs were lost due fungal contamination. Thirty-seven isolates were identified as *Steinernema* and the remaining 43 isolates were identified as *Heterorhabditis*. *Steinernema* (35 isolates) were identified as *S*. *surkhetense* (97–99% identity). One isolate was identified as *S*. *sangi* with 98% similarity. Species of the remaining one isolate *Steinernema* eKK26.2_TH was unidentified due to its low identity with *S*. *abbasi* (90%).

**Fig 4:**
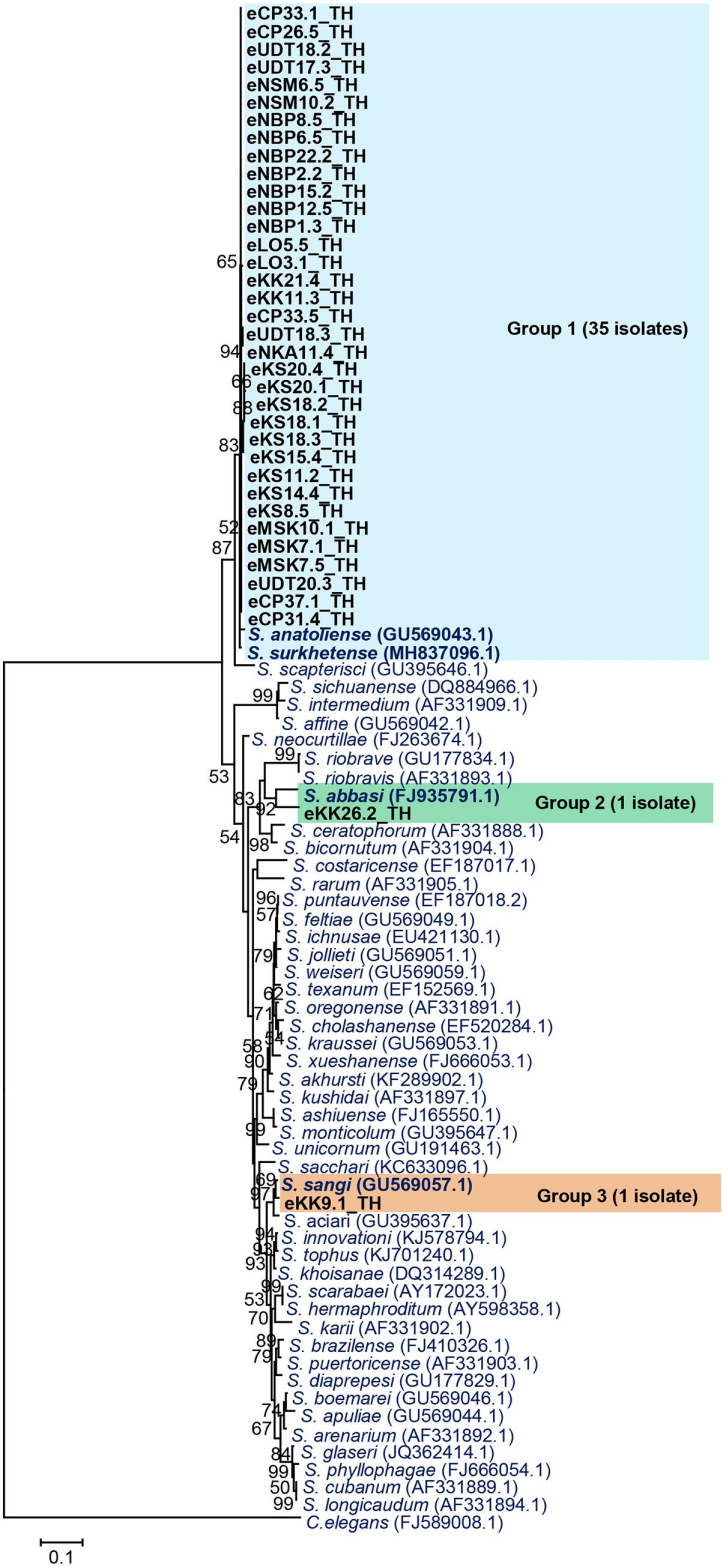
Maximum likelihood tree of 37 *Steinernema* isolates (black bold letter) based on the partial sequence of 28S rDNA (623–630 bp) compared with *Steinernema* reference strains from NCBI. *Caenorhabditis elegans* was used as an out-group. Bootstrap values are reported out of 1000 replicates. Numbers shown above the branches are bootstrap percentages for clades supported above the 50% level. The bar indicates 10% sequence divergence.

The phylogenetic relationships among the *Steinernema* isolates and reference strains from GenBank database are shown in Fig 4. The ML analysis of the 37 sequences distributed them into three groups. Group 1 contained 35 isolates of *Steinernema*, which were closely related to *S*. *surkhetense* and *S*. *anatoliense*. Group 2 contained only one isolate, which was closely related to *S*. *abbasi*. Group 3 contained one isolate of *Steinernema*, which was closely related to *S*. *sangi*.

**Fig 5:**
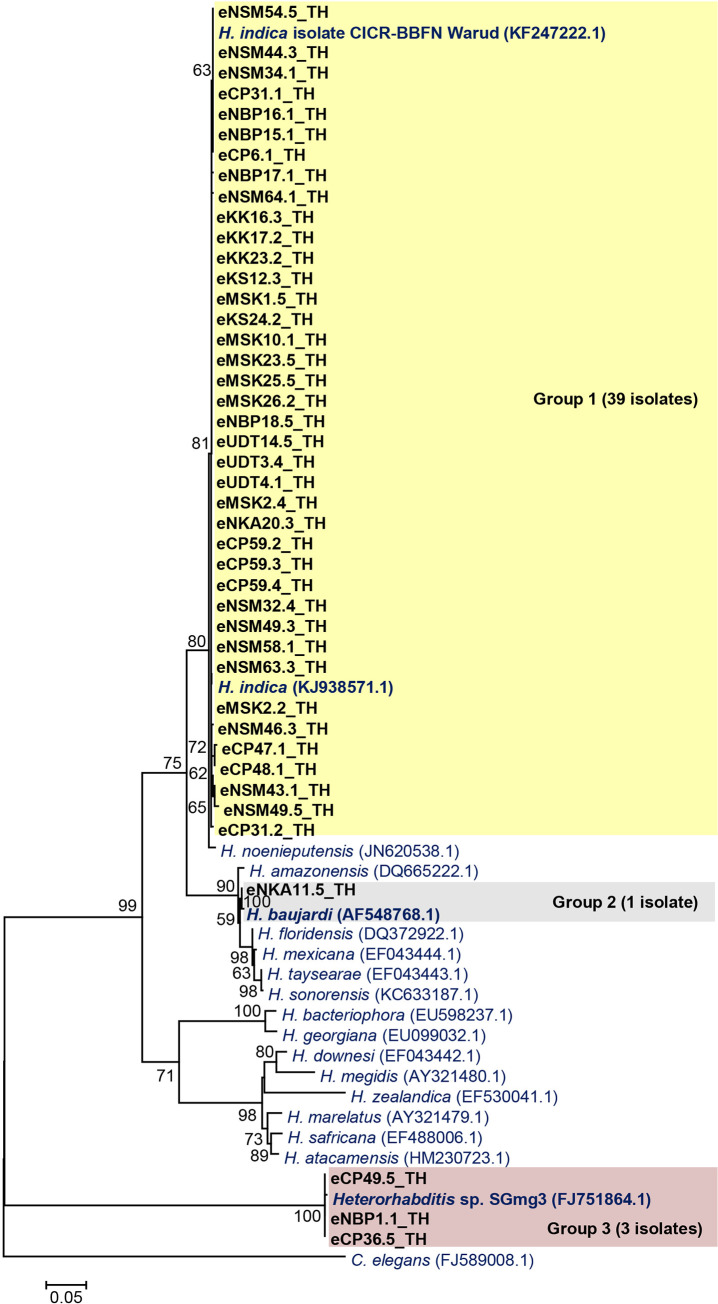
Maximum likelihood tree of 43 *Heterorhabditis* isolates (black bold letter) based on the partial sequence of internal transcribed spacer (495–548 bp) compared with *Heterorhabditis* reference strains from NCBI. *Caenorhabditis elegans* was used as an out-group. Bootstrap values are reported out of 1000 replicates. Numbers shown above the branches are bootstrap percentages for clades supported above the 50% level. The bar indicates 5% sequence divergence.

For *Heterorhabditis* nematodes, 39 isolates were identified as *H*. *indica* (98–100% identity), one isolate was identified as *H*. *baujardi* (99% identity), and three isolates were identified as *Heterorhabditis* sp. SGmg3 (97–99% identity). ML analysis of the 43 sequences of *Heterorhabditis* distributed them into three groups (Fig 5). Group 1 was the majority group (39 isolates), which was closely related to the clade of *H*. *indica*. Group 2 contained only one isolate, which was closely related to *H*. *baujardi*. Group 3 contained three isolates, which were closely related to *Heterorhabditis* sp. SGmg3.

Maximum association was observed between *X*. *stockiae* and the nematode host *S*. *surkhetense* (35 isolates). A single isolate of *X*. *indica* was associated with *Steinernema* sp., and one isolate of *Xenorhabdus* sp. (bKK9.1_TH), closely related to *X*. *eapokensis*, was associated with *S*. *sangi*. In addition, 39 isolates of *P*. *luminescens* were associated with *H*. *indica*. A single isolate of *P*. *luminescens* subsp. *akhurstii* was associated with *H*. *baujardi*. Two isolates of *P*. *asymbiotica* subsp. *australis* were associated with *H*. *indica*. A single isolate of *P*. *luminescens* subsp. *luamondii* was associated with *Heterorhabditis* sp. SGmg3.

### Antibacterial activity

We found that whole cell extracts of four (*X*. *stockiae*, n = 3 and *X*. *indica*, n = 1) out of 113 isolates could inhibit the growth of at least one antibiotic-resistant bacterial strain. The extract from these bacterial isolates was tested against the antibiotic-resistant bacteria by disk diffusion method. Two isolates of *X*. *stockiae* (bMSK7.5_TH and bKS8.5_TH) and one isolate of *X*. *indica* (bKK26.2_TH) showed potential inhibition of the growth of the antibiotic-resistant bacteria (Table 3). *X*. *stockiae* (bMSK7.5_TH) could inhibit *A*. *baumannii* strain AB320 (extensively drug resistant; XDR), *A*. *baumannii* strain AB321, AB322 (multi drug resistant; MDR), *A*. *baumannii* strain AB324 (XDR), *S*. *aureus* ATCC20475, *S*. *aureus* strain PB36 (methicillin resistance *Staphylococcus aureus*; MRSA), *E*. *coli* ATCC35218, *E*. *coli* strain PB1 (extended spectrum beta-lactamase; ESBL and MDR), *E*. *coli* strain PB231 (ESBL and carbapenem-resistant *Enterobacteriaceae*; CRE), *P*. *aeruginosa* strain PB30 (MDR), *K*. *pneumoniae* ATCC700603, *K*. *pneumoniae* strain PB5 (ESBL and MDR), and *K*. *pneumoniae* strain PB21 (ESBL and CRE). *X*. *stockiae* (bKS8.5_TH) and *X*. *indica* (bKK26.2_TH) could inhibit *S*. *aureus* strain PB36 (MRSA). However, *X*. *stockiae* (bUDT18.2_TH) was unable to inhibit any antibiotic-resistant bacteria by the disk diffusion method.

**Table 3:** Antibacterial activity of *Xenorhabdus* and *Photorhabdus* extracts against antibiotic-resistant bacteria as assessed by disk diffusion.

Bacteria (code)	Inhibit the growth of drug resistant bacteria														
	*A*. *baumannii* strain AB320 (XDR) ^a^	*A*. *baumannii* strain AB321 (MDR) ^b^	*A*. *baumannii* strain AB322 (MDR) ^b^	*A*. *baumannii* strain AB324 (XDR) ^a^	*S*. *aureus* ATCC20475	*S*. *aureus* strain PB36 (MRSA)^c^	*S*. *aureus* strain PB57 (MRSA)^c^	*E*. *coli* ATCC35218	*E*. *coli* strain PB1 (ESBL and MDR)^d^^,^^b^	*E*. *coli* strain PB231 (ESBL and CRE) ^d^^,e^	*P*. *aeruginosa* strain PB30 (MDR)^b^	*E*. *faecalis* ATCC51299	*K*.*pneumoniae*ATCC700603	*K*. *pneumoniae* strain PB5 (ESBL and MDR) ^d^^,^^b^	*K*. *pneumoniae* strain PB21 (ESBL and CRE) ^d^^,e^
*X*. *stockiae* (bMSK7.5_TH)	+	+	++	+	++	++	-	+	+	+	+	-	+	+	+
*X*. *stockiae* (bKS8.5_TH)	-	-	-	-	-	+	-	-	-	-	-	-	-	-	-
*X*. *stockiae* (bUDT18.2_TH)	-	-	-	-	-	-	-	-	-	-	-	-	-	-	-
*X*. *indica* (bKK26.2_TH)	-	-	-	-	-	+	-	-	-	-	-	-	-	-	-

- No activity (6 mm), + weak inhibition (7–10 mm.), ++ moderate/average inhibition (11–15 mm.)

^a^extensively drug resistant

^b^multidrug resistant

^c^methicillin resistance *Staphylococcus aureus*, and

^d^extended spectrum beta-lactamase, Carbapenem-resistant *Enterobacteriaceae*

We also evaluated the MIC and MBC of *X*. *stockiae* (bMSK7.5_TH) extract against 13 antibiotic-resistant bacterial strains, including *A*. *baumannii* strain AB320 (XDR), *A*. *baumannii* strain AB321, AB322 (MDR), *A*. *baumannii* strain AB324 (XDR), *S*. *aureus* ATCC20475, *S*. *aureus* strain PB36 (MRSA), *E*. *coli* ATCC35218, *E*. *coli* strain PB1 (ESBL and MDR), *E*. *coli* strain PB231 (ESBL and CRE), *P*. *aeruginosa* strain PB30 (MDR), *K*. *pneumoniae* ATCC700603, *K*. *pneumoniae* strain PB5 (ESBL and MDR), and *K*. *pneumoniae* strain PB21 (ESBL and CRE). MIC and MBC of *X*. *stockiae* (bMSK7.5_TH) extract against these antibiotic-resistant bacteria were 3.90 mg/mL and 7.81 mg/mL, respectively, whereas *X*. *stockiae* (bKS8.5_TH) and *X*. *indica* (bKK26.2 TH) showed potential efficacy only against *S*. *aureus* strain PB36 (MRSA). MIC and MBC of *X*. *stockiae* (bKS8.5_TH) and *X*. *indica* (bKK26.2_TH) extracts against *S*. *aureus* strain PB36 were 62.5 mg/mL and 15.62 mg/mL, respectively (Table 4).

**Table 4:** Antibacterial activity of *Xenorhabdus* extracts against antibiotic-resistant bacteria as assessed by minimum inhibitory concentration and minimal bactericidal concentration.

Bacterial list (Code)	Concentration of inhibition (mg/mL)									
	*S*. *aureus* strain PB36 (MRSA)		*K*. *pneumoniae* strain PB5 (ESBL+MDR)		*A*. *baumannii* strain AB320 (XDR)		*P*. *aeruginosa* strain PB30 (MDR)		*E*. *coli* strain PB1 (ESBL+MDR)	
	MIC	MBC	MIC	MBC	MIC	MBC	MIC	MBC	MIC	MBC
*X*. *stockiae* (bMSK7.5_TH)	3.9	7.81	3.9	7.81	3.9	7.81	3.9	7.81	3.9	7.81
*X*. *stockiae* (bKS8.5_TH)	62.5	62.5	-	-	-	-	-	-	-	-
*X*. *indica* (bKK26.2_TH)	15.62	15.62	-	-	-	-	-	-	-	-

## Discussion

The overall recovery rate of the EPNs (*Steinernema* and *Heterorhabditis*) from soil samples of Northeastern region of Thailand was 7.82%. This result was similar to that reported by Brodie [31] from Fiji Islands (7.3%), Valadas [32] from Portugal (6.7%), and Hatting [33] from South Africa (5%). However, this rate was higher than those reported by Caoili [34] from the Philippines (2.5%), Majić [35] from Croatia (2.0%), and Noujeim [36] from Lebanon (1%). Higher prevalence of EPNs in soil from that determined in the present study was observed by Kanga [37] from Southern Cameroon (10.4%), Khatri-Chhetri [38] in Nepal (10.5%), and Malan [39] in South Africa (17%). This suggests that global prevalence of EPNs is variable. Distribution of *Steinernema* and *Heterorhabditis* has been reported from several ecological niches in USA, Australia, Europe, and Asia, including Thailand [8, 15–18, 24, 40, 41]. Biotic and abiotic characteristics influence the distribution of the EPNs; however, in our study, soil temperature, moisture, and pH of the soil samples with and without EPNs were not significantly different. Nevertheless, our data supported previous reports from Thailand, which showed that EPNs were able to survive in a diverse soil environment and various soil types with a wide range of pH (3.2–6.9), temperature (20°C-32°C), and moisture (0–8%) [17, 18, 22, 23]. Soil moisture, temperature, and rainfall affect the distribution of the insects that could be probable hosts for the EPNs [42]. This could also affect the distribution of EPNs.

Identification and phylogenetic analysis of 62 *Xenorhabdus* isolates revealed that *X*. *stockiae* was the predominant species. This bacterium was hosted by *S*. *surkhetense*, which has been previously described from India [43]. *X*. *stockiae* has also been isolated from *S*. *siamkayai* and *S*. *minutum* in Thailand [16, 24, 44, 45]. It was reported as a bacterial symbiont with *S*. *huense* in Vietnam [46]. One isolate of *X*. *indica* was found to be associated with *Steinernema* sp. (90% similar with *S*. *abbasi*). *X*. *indica* was first reported to be associated with *S*. *thermophilum* [47]. Subsequently, the association between *X*. *indica* and *S*. *abbasi* was reported from Taiwan [48]. In a previous study, *X*. *indica* was associated with *S*. *yirgalemense* [49], and in the current study, it was found that *X*. *indica*, an Indian isolate, was associated with *S*. *pakistanense* [50]. This suggests that *X*. *indica* may be symbiotically associated with a wide range of EPN hosts.

A single isolate of *Xenorhabdus* (bKK9.1_TH) showed low similarity with *X*. *thuongxuanensis* (95% identity) and *X*. *eapokensis* (95% identity) by *recA* sequence analysis. In contrast, higher similarity of this isolate was found with *X*. *eapokensis* when 16S rDNA and *infB* (99% identity), and *gltX* and *dnaN* (98% identity) sequences were analyzed. In addition, multilocus sequence analysis (MLSA) based on concatenated partial gene sequences of *recA*, *gyrB*, *dnaN*, *gltX*, and *infB* revealed that *Xenorhabdus* (bKK9.1_TH) was closely related to *X*. *eapokensis*. Therefore, we identified *Xenorhabdus* (bKK9.1_TH) as *X*. *eapokensis*. This suggests that analysis of multiple genes may aid in the identification of this bacterium. Also, whole genome sequencing of this bacterium may assist in confirmation of its identity at the species level. We found that *X*. *eapokensis* was associated with *S*. *sangi*, which has been reported as a host for *X*. *vietnamensis* and *X*. *thuongxuanensis* [25, 51].

For the genus *Photorhabdus*, in the current study, the following four species were identified: *P*. *luminescens* subsp. *akhurstii* (n = 29), *P*. *luminescens* subsp. *hainanensis* (18 isolates), *P*. *luminescens* subsp. *luamondii* (n = 2), and *P*. *asymbiotica* subsp. *australis* (n = 2). *P*. *luminescens* subsp. *akhurstii* and *P*. *luminescens* subsp. *hainanensis* were associated with *H*. *indica* and *H*. sp. SGmg3. These associations have been previously reported from Thailand [15, 17, 18]. In addition, *P*. *luminescens* subsp. *hainanensis* has also been isolated from *H*. *baujardi* in Thailand [8]; however, *P*. *luminescens* subsp. *akhurstii* has been found in association with *H*. *bacteriophora* in Iran, Hungary, Argentina, USA, and in association with *H*. *indica* in China [19]. To the best of our knowledge, this is the first report on mutualistic association between *P*. *luminescens* subsp. *luamondii* and *Heterorhabditis* sp. SGmg3. However, *P*. *luminescens* subsp. *luamondii* has also been reported to be associated with *H*. *bacteriophora* in Thailand, USA, and Argentina [17, 52] and with *H*. *safricana* from South Africa [53]. *P*. *asymbiotica* subsp. *australis* was also found in the present study, which was in association with *H*. *indica*. This association has been found in Thailand previously [17, 54]. *P*. *asymbiotica* is an emerging pathogen that has been reported to cause locally invasive soft tissue infection and disseminated bacteremia; clinical cases have been identified in both Australia and USA [55, 56]. This suggests that *P*. *asymbiotica* could also cause these diseases in the residents of Thailand. Although no clinical case of *P*. *asymbiotica* infection has been reported in the country, management and healthcare strategies should be prepared in advance.

In the current study, *X*. *stockiae* (bMSK7.5_TH) extract showed the highest inhibitory effect against several antibiotic-resistant bacteria. Previous studies have shown that *Xenorhabdus* produces xenocoumacin [57] and amicoumacin derivatives [58], which are potent against *S*. *aureus* [59]. All *Photorhabdus* extracts from Mae Wong national park could inhibit *S*. *aureus* ATCC20475, *S*. *aureus* strain PB36 (MRSA), and *S*. *aureus* strain PB57 (MRSA) (8). In addition, *P*. *luminescens* subsp. *akhurstii* (bSBR11.1_TH) extract from Saraburi province could inhibit up to 10 antibiotic-resistant bacterial strains, and all *Photorhabdus* isolates showed the potential to inhibit the growth of *S*. *aureus* strain PB36 (MRSA) [41]. *P*. *luminescens* has been reported to produce isopropylstilbene [44, 60], which has multiple biological activities, including antibiotic activity against *E*. *coli*, *B*. *subtilis*, *S*. *pyogenes*, and *S*. *aureus* RN4220 (drug resistant and clinical isolate) [61, 62]. The bio-activity of isopropylstilbene has been extended to inhibit the growth of fungi [10]. We found that the bioactivity (MIC and MBC) of the crude extracts emphasized that the *X*. *stockiae* (bMSK7.5_TH) extracts were active against both gram-positive and gram-negative bacteria. The MIC values exhibited by all the extracts in this study ranged between 3.90–62.5 mg/mL, and the MBC ranged between 7.81–15.62 mg/ml. This may be due to the ability of each symbiotic bacterial isolate to produce different effective metabolites to kill drug resistant bacteria. This suggests that *Xenorhabdus* and *Photorhabdus* isolates are potential agents for the inhibition of the growth of MDR bacteria. Therefore, both *Xenorhabdus* and *Photorhabdus* isolates are a potential source for novel antibiotics.

## Conclusions

In summary, 113 isolates of EPNs were obtained from a total of 1,445 soil samples collected from 289 sites in Northeastern region of Thailand. *S*. *surkhetense* and *H*. *indica* were the two most common EPN species found in the soil samples. For symbiotic bacteria, *X*. *stockiae*, *X*. *indica*, *X*. *eapokensis*, *P*. *luminescens* subsp. *akhurstii*, *P*. *luminescens* subsp. *hainanensis*, and *P*. *asymbiotica* subsp. *australis* were found in the studied area, and *X*. *stockiae* and *P*. *luminescens* subsp. *akhurstii* were found to be predominant. The common associations observed between EPN hosts and their symbiotic bacteria were *S*. *surkhetense-X*. *stockiae* and *H*. *indica*-*P*. *luminescens*. EPN host of *X*. *eapokensis* was *S*. *sangi* and that of *X*. *indica* was unidentified *Steinernema*. In addition, the crude extract from *X*. *stockiae* (bMSK7.5_TH) showed a broad-spectrum inhibitory activity against several antibiotic-resistant bacterial strains. Thus, this study will be useful in further drug discovery from natural resources.

## Supporting information

S1 Fig Maximum likelihood phylogenetic tree of *Xenorhabdus* (KK9.1_TH) based on a partial 16S rDNA sequence (1,401 bp) compared with *Xenorhabdus* strains downloaded from GenBank. *P*. *luminescens* subsp. *luminescens* was used as an out-group. Bootstrap values are reported out of 1000 replicates. The numbers shown above the branches are support values of Maximum likelihood/Neighbor-joining/Bayesian posterior probabilities for clades supported above the 50% level. The bar indicates 5% sequence divergence.
(DOCX)

S2 Fig Maximum likelihood phylogenetic tree of *Xenorhabdus* (KK9.1_TH) based on a partial *gyrB* sequence (846 bp) compared with *Xenorhabdus* strains downloaded from GenBank. *P*. *luminescens* subsp. *luminescens* was used as an out-group. Bootstrap values are reported out of 1000 replicates. The numbers shown above the branches are support values of Maximum likelihood/Neighbor-joining/Bayesian posterior probabilities for clades supported above the 50% level. The bar indicates 2% sequence divergence.
(DOCX)

S3 Fig Maximum likelihood phylogenetic tree of *Xenorhabdus* (KK9.1_TH) based on a partial *dnaN* sequence (828 bp) compared with *Xenorhabdus* strains downloaded from GenBank. *P*. *luminescens* subsp. *luminescens* was used as an out-group. Bootstrap values are reported out of 1000 replicates. The numbers shown above the branches are support values of Maximum likelihood/Neighbor-joining/Bayesian posterior probabilities for clades supported above the 50% level. The bar indicates 5% sequence divergence.
(DOCX)

S4 Fig Maximum likelihood phylogenetic tree of *Xenorhabdus* (KK9.1_TH) based on a partial *gltX* sequence (1,057 bp) compared with *Xenorhabdus* strains downloaded from GenBank. *P*. *luminescens* subsp. *luminescens* was used as an out-group. Bootstrap values are reported out of 1000 replicates. The numbers shown above the branches are support values of Maximum likelihood/Neighbor-joining/Bayesian posterior probabilities for clades supported above the 50% level. The bar indicates 2% sequence divergence.
(DOCX)

S5 Fig Maximum likelihood phylogenetic tree of *Xenorhabdus* (KK9.1_TH) based on a partial *infB* sequence (1,052 bp) compared with *Xenorhabdus* strains downloaded from GenBank. *P*. *luminescens* subsp. *luminescens* was used as an out-group. Bootstrap values are reported out of 1000 replicates. The numbers shown above the branches are support values of Maximum likelihood/Neighbor-joining/Bayesian posterior probabilities for clades supported above the 50% level. The bar indicates 2% sequence divergence.
(DOCX)
