# Supplementary material for: A study on Xenorhabdus and Photorhabdus isolates from Northeastern Thailand: Identification, antibacterial activity, and association with entomopathogenic nematode hosts
Source: PLoS One. 2021 Aug 12;16(8):e0255943. doi: 10.1371/journal.pone.0255943 (PMC8360611; doi:10.1371/journal.pone.0255943)
Supplement: S1 Fig — P. luminescens subsp. luminescens was used as an out-group. Bootstrap values are reported out of 1000 replicates. The numbers shown above the branches are support values of Maximum likelihood/Neighbor-joining/Bayesian posterior probabilities for clades supported above the 50% level. The bar indicates 5% sequence divergence. (DOCX) [file pone.0255943.s001.docx]

*X. thuongxuanensis* (NR 156924.1)

*X. ehlersii* (AJ810294.1)

*X. budapestensis* (AJ810293.1)

*X. kozodoii* (DQ211716.1)

*X. stockiae* (DQ202309.1)

*X. innexi* (AJ810292.2)

*X. hominickii* (AB507815.1)

*X. koppenhoeferi* (DQ205450.1)

*X. japonica* (D78008.1)

*X. indica* (DQ211718.1)

*X. indica* (AB507813.1)

*X. poinarii* (D78010.1)

*X. ishibashii* (AB243427.1)

*X. griffiniae* (DQ211710.1)

**KK9.1 TH**

*X. eapokensis* (NR 156925.1)

*X. miraniensis* (DQ211713.1)

*X. mauleonii* (DQ211715.1)

*X. szentirmaii* (AJ810295.1)

*X. nematophila* (D78009.1)

*X. romanii* (DQ211717.1)

*X. doucetiae* (DQ211709.1)

*X. cabanillasii* (AY521244.1)

*X. beddingii* (D78006.1)

*P. luminescens* subsp. *luminescens* (X82248.1)

99/100/100

78/99/98

78/97/81

77/64/-

69/87/-

63/67/62

55/69/58

0.005

**S1 Fig.** Maximum likelihood phylogenetic tree of *Xenorhabdus* (KK9.1 TH) based on a partial 16S rDNA sequence (1,401 bp) compared with *Xenorhabdus* strains downloaded from GenBank. *P. luminescens* subsp. *luminescens* was used as an out-group. Bootstrap values are reported out of 1000 replicates. The numbers shown above the branches are support values of Maximum likelihood/Neighbor-joining/Bayesian posterior probabilities for clades supported above the 50% level. The bar indicates 5% sequence divergence.
