# Supplementary material for: A study on Xenorhabdus and Photorhabdus isolates from Northeastern Thailand: Identification, antibacterial activity, and association with entomopathogenic nematode hosts
Source: PLoS One. 2021 Aug 12;16(8):e0255943. doi: 10.1371/journal.pone.0255943 (PMC8360611; doi:10.1371/journal.pone.0255943)
Supplement: S2 Fig — P. luminescens subsp. luminescens was used as an out-group. Bootstrap values are reported out of 1000 replicates. The numbers shown above the branches are support values of Maximum likelihood/Neighbor-joining/Bayesian posterior probabilities for clades supported above the 50% level. The bar indicates 2% sequence divergence. (DOCX) [file pone.0255943.s002.docx]

**KK9.1 TH**

*X. eapokensis* (KY451960.1)

*X. ishibashii* (AB630948.1)

*X. ehlersii* (EU934524.1)

*X. griffiniae* (EU934525.1)

*X. thuongxuanensis* (KY451961.1)

*X. kozodoii* (FJ533194.1)

*X.kozodoii* (EU934523.1)

*X. doucetiae* (FJ533192.1)

*X. magdalenensis* (JF798402.1)

*X. magdalenensis* (JF798402.1)

*X.miraniensis* (EU934520.1)

*X. khoisanae* (JX623982.1)

*X. innexi* (FJ533206.1)

*X. stockiae* (EU934542.1)

*X. cabanillasii* (FJ533205.1)

*X. budapestensis* (FJ533198.1)

*X. indica* (FJ533188.1)

*X. hominickii* (FJ533195.1)

*X. beddingii* (FJ533200.1)

*X. japonica* (EU934513.1)

*X. szentirmaii* (FJ533197.1)

*X. mauleonii* (EU934533.1)

*X. koppenhoeferi* (FJ533208.1)

*Photorhabdus luminescens* subsp. *luminescens* (AY278501.1)

99/100/100

100/100/-

100/100/-

100/100/100

98/100/100

93/95/97

96/99/100

78/99/98

70/-/82

90/99/99

85/78/100

90/95/99

94/93/99

57/80/79

97/96/100

0.02

**S2 Fig.** Maximum likelihood phylogenetic tree of *Xenorhabdus* (KK9.1 TH) based on a partial gyrB sequence (846 bp) compared with *Xenorhabdus* strains downloaded from GenBank. *P. luminescens* subsp. *luminescens* was used as an out-group. Bootstrap values are reported out of 1000 replicates. The numbers shown above the branches are support values of Maximum likelihood/Neighbor-joining/Bayesian posterior probabilities for clades supported above the 50% level. The bar indicates 2% sequence divergence.
