# Supplementary material for: A study on Xenorhabdus and Photorhabdus isolates from Northeastern Thailand: Identification, antibacterial activity, and association with entomopathogenic nematode hosts
Source: PLoS One. 2021 Aug 12;16(8):e0255943. doi: 10.1371/journal.pone.0255943 (PMC8360611; doi:10.1371/journal.pone.0255943)
Supplement: S3 Fig — P. luminescens subsp. luminescens was used as an out-group. Bootstrap values are reported out of 1000 replicates. The numbers shown above the branches are support values of Maximum likelihood/Neighbor-joining/Bayesian posterior probabilities for clades supported above the 50% level. The bar indicates 5% sequence divergence. (DOCX) [file pone.0255943.s003.docx]

**KK9.1 TH**

*X. eapokensis* (KX602189.1)

*X. ishibashii* (AB630949.1)

*X. thuongxuanensis* (KX602195.1)

*X. ehlersii* (FJ831448.1)

*X. griffiniae* (FJ831449.1)

*X. kozodoii* (FJ831447.1)

*X. doucetiae* (FJ831450.1)

*X. romanii* (FJ831451.1)

*X. magdalenensis* (JF798399.1)

*X. magdalenensis* (JF798399.1

*X.poinarii* (FJ831454.1)

*X. poinarii* (FJ831456.1)

*X. japonica* (FJ831453.1)

*X. beddingii* FJ831460.1)

*X. khoisanae* (JX623980.1)

*X. miraniensis* (FJ831459.1)

*X. hominickii* (FJ831463.1)

*X. mauleonii* (FJ831464.1)

*X. koppenhoeferi* (FJ831457.1)

*X. szentirmaii* (FJ831458.1)

*X. innexi* (FJ831476.1)

*X. stockiae* (FJ831477.1)

*X. indica* (FJ831471.1)

*X. cabanillasii* (FJ831472.1)

*X. budapestensis* FJ831474.1)

*P. luminescens* subsp. *luminescens* (FJ831501.1)

100/100/100

55/-/75

98/100/99

100/100/100

100/100/-

99/100/100

100/100/100

100/100/100

51/87/-

83/90/98

87/-/-

54/-/95

98/99/100

91/94/97

69/71/99

98/-/100

0.05

**S3 Fig.** Maximum likelihood phylogenetic tree of *Xenorhabdus* (KK9.1 TH) based on a partial dnaN sequence (828 bp) compared with *Xenorhabdus* strains downloaded from GenBank. *P. luminescens* subsp. *luminescens* was used as an out-group. Bootstrap values are reported out of 1000 replicates. The numbers shown above the branches are support values of Maximum likelihood/Neighbor-joining/Bayesian posterior probabilities for clades supported above the 50% level. The bar indicates 5% sequence divergence.
