# Supplementary material for: A study on Xenorhabdus and Photorhabdus isolates from Northeastern Thailand: Identification, antibacterial activity, and association with entomopathogenic nematode hosts
Source: PLoS One. 2021 Aug 12;16(8):e0255943. doi: 10.1371/journal.pone.0255943 (PMC8360611; doi:10.1371/journal.pone.0255943)
Supplement: S4 Fig — P. luminescens subsp. luminescens was used as an out-group. Bootstrap values are reported out of 1000 replicates. The numbers shown above the branches are support values of Maximum likelihood/Neighbor-joining/Bayesian posterior probabilities for clades supported above the 50% level. The bar indicates 2% sequence divergence. (DOCX) [file pone.0255943.s004.docx]

**KK9.1 TH**

*X. eapokensis* (KX602190.1)

*X. thuongxuanensis* (KX602196.1)

*X. ishibashii* (AB630950.1)

*X. griffiniae* (FJ840496.1)

*X. ehlersii* (FJ840495.1)

*X. kozodoii* (FJ840494.1)

*X. romanii*(FJ840498.1)

*X. doucetiae* (FJ840497.1)

*X. magdalenensis* (JF798400.1)

*X. magdalenensis* (JF798400.1

*X. japonica* (FJ840503.1)

*X. poinarii* (FJ840501.1)

*X. poinarii* (FJ840499.1)

*X. koppenhoeferi* (FJ840504.1)

*X. beddingii* (FJ840506.1)

*X. miraniensis* (FJ840505.1)

*X. khoisanae* (JX623981.1)

*X. mauleonii* (FJ840507.1)

*X. szentirmaii* (FJ840508.1)

*X. hominickii* (FJ840510.1)

*X. stockiae* (FJ840524.1)

*X. cabanillasii* (FJ840521.1)

*X. indica* (FJ840520.1)

*X. budapestensis* (FJ840518.1)

*X. innexi* (FJ840523.1)

*P. luminescens* subsp. *luminescens* (FJ844912.1)

99/100/-

90/77/96

99/100/100

100/100/-

99/100/100

100/100/100

99/100/100

100/100/100

51/81/55

63/91/73

77/65/98

97/-/100

72/98/90

82/79/100

80/55/99

79/95/99

58/82/-

0.02

**S4 Fig.** Maximum likelihood phylogenetic tree of *Xenorhabdus* (KK9.1 TH) based on a partial gltX sequence (1,057 bp) compared with *Xenorhabdus* strains downloaded from GenBank. *P. luminescens* subsp. *luminescens* was used as an out-group. Bootstrap values are reported out of 1000 replicates. The numbers shown above the branches are support values of Maximum likelihood/Neighbor-joining/Bayesian posterior probabilities for clades supported above the 50% level. The bar indicates 2% sequence divergence*.*
