# Supplementary material for: A study on Xenorhabdus and Photorhabdus isolates from Northeastern Thailand: Identification, antibacterial activity, and association with entomopathogenic nematode hosts
Source: PLoS One. 2021 Aug 12;16(8):e0255943. doi: 10.1371/journal.pone.0255943 (PMC8360611; doi:10.1371/journal.pone.0255943)
Supplement: S5 Fig — P. luminescens subsp. luminescens was used as an out-group. Bootstrap values are reported out of 1000 replicates. The numbers shown above the branches are support values of Maximum likelihood/Neighbor-joining/Bayesian posterior probabilities for clades supported above the 50% level. The bar indicates 2% sequence divergence. (DOCX) [file pone.0255943.s005.docx]

**KK9.1 TH**

*X. eapokensis* (KX602192.1)

*X. griffiniae* (JF798405.1)

*X. ehlersii* (JF798404.1)

*X. thuongxuanensis* (KX602198.1)

*X. ishibashii* (AB690831.1)

*X. kozodoii* (JF798411.1)

*X. magdalenensis* (JF798403.1)

*X. romanii* (JF798407.1)

*X. doucetiae* (JF798406.1)

*X. japonica* (JF798409.1)

*X. vietnamensis* (JF798408.1)

*X. poinarii* (JF798410.1)

*X. beddingii* (JF798413.1)

*X. miraniensis* (JF798412.1)

*X. khoisanae* (JX623984.1)

*X. hominickii* (JF798414.1)

*X. koppenhoeferi* (JF798417.1)

*X. mauleonii* (JF798415.1)

*X. szentirmaii* (JF798416.1)

*X. stockiae* (JF798423.1)

*X. innexi* (JF798422.1)

*X. indica* (JF798421.1)

*X. budapestensis* (JF798419.1)

*X. cabanillasii* (JF798420.1)

*P. luminescens* subsp. *luminescens* (JQ901852.1)

68/84/88

99/100/100

99/100/100

75/87/93

97/98/97

100/100/100

99/99/100

100/99/100

94/97/100

57/-/96

99/100/100

91/96/100

51/-/51

82/85/99

99/99/100

91/88/86

73/73/-

83/84/99

65/52/88

0.02

**S5 Fig.** Maximum likelihood phylogenetic tree of *Xenorhabdus* (KK9.1 TH) based on a partial infB sequence (1,052 bp) compared with *Xenorhabdus* strains downloaded from GenBank. *P. luminescens* subsp. *luminescens* was used as an out-group. Bootstrap values are reported out of 1000 replicates. The numbers shown above the branches are support values of Maximum likelihood/Neighbor-joining/Bayesian posterior probabilities for clades supported above the 50% level. The bar indicates 2% sequence divergence.
